# Exploring equine therapy for people with dementia in long‐term care homes

**DOI:** 10.1002/alz70858_102189

**Published:** 2025-12-25

**Authors:** Marie‐Lee Yous, Megan Campbell, Brian Kim, Irene Hartigan, Christa Sedore, Jennifer Semach, Sathana Surendran, Amanda Tran, Jessie Xu, Sharon Kaasalainen

**Affiliations:** ^1^ McMaster University, Hamilton, ON, Canada; ^2^ University College Cork, Cork, Cork, Ireland; ^3^ Walkabout Farm Therapeutic Riding Association Inc, Minden, ON, Canada

## Abstract

**Background:**

Equine(horse)‐therapy or equine‐assisted activities have been found to reduce stress, improve quality of life, and promote autonomy for people with dementia in long term care (LTC). In 2024, the [Walkabout Farm Therapeutic Riding Association Inc] in Ontario, Canada, recently launched a new program called R.E.A.P. (Recreational Equine Assisted Participaction). This program provides older adults in LTC homes full sensory engagement through interactions with horses, including petting, grooming, walking and feeding. Sessions, led by trained facilitators, last approximately 45 minutes and are hosted indoors at LTC homes, or on‐site at Walkabout Farm. To date, no evaluation of equine‐assisted activities for people with dementia living in LTC exists in Canada. The purpose of this study is to explore the feasibility, acceptability, and observed effects R.E.A.P. in LTC.

**Method:**

The research design consists of qualitative description with quantitative data collected in terms of feasibility (i.e., number of sessions provided per resident per week, length of sessions) and observed effects (i.e., engagement with the horses/stimuli; interactions between residents, caregivers, and staff). Research staff collected feasibility and observation data during the R.E.A.P. sessions. Data collection occurred in person either at the Walkabout Farm or at the LTC home. Interviews with residents and staff occurred in person, by phone or by videoconferencing (i.e., Zoom). Descriptive statistics were used for quantitative statistics and direct content analysis for the interview data.

**Result:**

A total of 12 participants completed the study including 3 staff members and 9 residents. None of the participants refused to participate in the sessions. Out of the 34 sessions delivered, residents were very attentive to attentive towards the horses (79%) and talked with the horses during the sessions (88%). Findings from the interviews revealed that sessions helped to foster relationships between residents, the horses, and staff, created emotional connection for residents, elicited enjoyment, and promoted reminiscing.

**Conclusion:**

The R.E.A.P. program demonstrated meaningful benefits to residents in LTC, including enhanced emotional well‐being, social engagement and quality of life. These findings highlight the potential of equine therapy in enriching and supporting the well‐being of residents in LTC.

